# Before the Gut: Human Milk Oligosaccharides and Oral Microbial Colonization in Infancy

**DOI:** 10.3390/nu18193269

**Published:** 2026-10-04

**Authors:** Oana-Raluca Temneanu, Felicia Trofin, Adriana Mihai, Ancuța Lupu, Raluca Olariu, Manuela Ștefan, Elena-Roxana Buzilă, Otilia Novac, Roxana Șerban, Vasile Valeriu Lupu, Emil Anton, Mihaela Oros, Bianca Simionescu

**Affiliations:** 1 Grigore T. Popa University of Medicine and Pharmacy, 700115 Iași, Romania; temneanu.oana@umfiasi.ro (O.-R.T.); ancuta.ignat1@umfiasi.ro (A.L.); raluca.olariu@umfiasi.ro (R.O.); stefan.manuela@umfiasi.ro (M.Ș.); elena-roxana.buzila@umfiasi.ro (E.-R.B.); otilia.novac@umfiasi.ro (O.N.); roxana-n-serban@umfiasi.ro (R.Ș.); vasile.lupu@umfiasi.ro (V.V.L.); emil.anton@umfiasi.ro (E.A.); 2 Titu Maiorescu University, 031593 Bucharest, Romania; mihaela.oros@prof.utm.ro; 3 Iuliu Hațieganu University of Medicine and Pharmacy, 400012 Cluj-Napoca, Romania; bianca.simionescu@umfcluj.ro

**Keywords:** human milk oligosaccharides, infant oral microbiome, secretor status, *FUT2*, dental biofilm, breastfeeding, early childhood caries, oral–gut axis

## Abstract

Background/Objectives: Human milk oligosaccharides (HMOs) reach the oral cavity before any other compartment and resist hydrolysis by salivary enzymes, yet their behavior there has received far less attention than their intestinal fate. This review asks what follows from treating the mouth as a compartment rather than as a corridor. Methods: MEDLINE, Scopus and Web of Science were searched from inception to August 2026, combining oligosaccharide and oral terms, with forward citation tracking of the human studies. Each candidate mechanism was classified by its type of supporting evidence: human oral association, direct oral in vitro evidence, indirect evidence from other compartments, or unresolved hypothesis. Results: Only two human analyses, both from the same Western Australian cohort (BLOSOM), have related HMO exposure to infant oral bacterial communities; the larger concluded that the effect, while statistically detectable, was biologically limited. Residence time, and probably effective glycan concentration, differ from the intestinal case, as do the aerotolerant, *Gemella*- and *Rothia*-dominated community and the salivary films coating teeth and mucosa. In vitro, 6′-sialyllactose and free *N*-acetylneuraminic acid inhibited *Streptococcus mutans* biofilm formation, but only at concentrations (~100 mM) well above plausible oral exposure; 2′-fucosyllactose had no antibiofilm effect, reduced adhesion in two of three strains and did not support growth. Maternal *FUT2* genotype shapes the oligosaccharide profile of milk, whereas the infant’s own *FUT2* genotype governs fucosylation of the salivary glycoproteins those oligosaccharides encounter; neither has been measured in infant oral microbiome studies. Conclusions: Anti-adhesive and substrate-driven mechanisms may operate in parallel, and a possible asymmetry between commensal and cariogenic streptococci is framed as a testable hypothesis, as is the contribution of swallowed oral communities to gut colonization. Current evidence supports no change in practice regarding supplemented formula, donor milk, oropharyngeal colostrum, or breastfeeding and caries.

## 1. Introduction

Human milk oligosaccharides (HMOs) constitute the third most abundant solid fraction of human milk, present at approximately 20–25 g/L in colostrum and 5–20 g/L in mature milk [1,2]. More than two hundred distinct structures have been characterized, each assembled on a lactose core extended by galactose, *N*-acetylglucosamine, fucose or *N*-acetylneuraminic acid (Neu5Ac) [1,3]. They are largely resistant to digestion by human gastrointestinal enzymes [4,5]. That apparent metabolic extravagance—a lactating woman synthesizing, at appreciable energetic cost, a class of molecules her child can barely use as a direct substrate—has driven two decades of inquiry into their biological purpose.

Almost all of that inquiry concerns the intestine. HMOs serve as selective growth substrates for Bifidobacterium, particularly *B. longum* subsp. *infantis*, whose glycan-foraging apparatus has been characterized in detail [6,7]; they shape the strength of priority effects during bifidobacterial community assembly [8,9]; they modulate epithelial and immune cell activity [10,11,12]; and they impede pathogen adhesion to the intestinal mucosa [13]. The corresponding literature is dense enough to sustain scoping reviews of its own [14].

The oral cavity has attracted less attention, although it is the first site HMOs reach and the one at which their structural profile most closely resembles that of the milk itself. Direct human evidence relating HMO exposure to the infant oral microbiome is limited to two analyses from a single Western Australian cohort, reviewed in Section 6.

Two studies from one research group in one geographic setting—with an empirical base so narrow, a synthesis must proceed mechanistically rather than evidentially.

The exercise is warranted for reasons that are partly clinical and partly conceptual. Oral bacterial profiles discriminate breastfed from formula-fed infants [15,16,17], and the early oral community is now understood to influence not only dental outcomes but a broader range of conditions in which oral organisms are implicated at distant sites [18,19,20,21,22,23,24]. Formulae supplemented with 2′-fucosyllactose and lacto-*N*-neotetraose are marketed at scale, yet their effect on oral colonization has never been assessed [25,26]. In neonatal intensive care, oropharyngeal administration of colostrum is increasingly used on the premise that its bioactive fraction acts locally, a premise examined at the level of the oral microbial community in only one small observational study [27].

Work performed in vitro offers a partial foothold. Sialylated HMOs and free Neu5Ac inhibit biofilm formation by *Streptococcus mutans* and by mixed streptococcal cultures at high concentrations [28], and milk oligosaccharides interfere with the attachment of other mucosal pathogens [29,30]. Older investigations established that human milk constituents reduce the adhesion of mutans streptococci to saliva-coated hydroxyapatite [31] and modify streptococcal biofilm formation [32]. The pattern that emerges is structure-dependent, and the structures that matter in the mouth need not be the ones that matter in the colon.

The aim of this review is to examine whether the infant oral cavity can be treated as a distinct compartment of HMO action rather than a transit route. To that end, we compare its physiology with that of the colon, classify the human, in vitro and extrapolated evidence for each candidate mechanism while keeping these levels of evidence separate, and state what current evidence does and does not support for infant feeding practice. Our working hypothesis, which the evidence reviewed here can refine but not confirm, is that any oral effects of HMOs operate mainly through structure-dependent interference with bacterial binding and species-selective substrate use, and that they are conditioned by two genotypes, maternal and infant *FUT2*, rather than one. A recent review from our group examined the anti-infective properties of HMOs more broadly [33]; the present work differs in scope, being restricted to the oral compartment, to commensal as well as pathogenic colonization, and to the clinical questions this compartment raises.

## 2. Materials and Methods

This work is a narrative review. No protocol was registered, no risk-of-bias appraisal was undertaken, and the synthesis is interpretive rather than quantitative. The search strategy is reported so that the reader may judge the coverage of the evidence considered. It was designed to be comprehensive for the question addressed but does not claim the exhaustiveness of a systematic review, and statements in the text about the number of available studies refer to studies identified by this search.

MEDLINE (via PubMed), Scopus and Web of Science Core Collection were searched from inception to August 2026. No lower date limit was applied, since several foundational adhesion studies predate the year 2000. Terms were combined across two blocks. The first comprised *human milk oligosaccharide*, *HMO*, *2′-fucosyllactose*, *3-fucosyllactose*, *sialyllactose*, *fucosyllactose*, *lacto-N-tetraose* and *lacto-N-neotetraose*. The second comprised *oral microbiome*, *oral microbiota*, *saliva*, *salivary pellicle*, *dental biofilm*, *dental plaque*, *Streptococcus mutans*, *mutans streptococci*, *early childhood caries* and *oral cavity*. Blocks were intersected with AND and terms within blocks with OR; MeSH descriptors were used where available, supplemented by free-text searching of titles and abstracts.

Records were restricted to English-language publications. Eligible material comprised human observational and interventional studies, animal studies and in vitro work addressing any interaction between milk oligosaccharides and oral bacterial species or communities. Reviews and commentaries were retained for context and reference harvesting but not treated as primary evidence. Reference lists of retrieved full texts were examined manually, and forward citation tracking was performed for the two human studies of HMO exposure and infant oral bacterial profiles. Forward citation tracking was repeated at revision and identified a further analysis from the same cohort relating non-oligosaccharide milk components to the infant oral microbiome [34]; it did not meet the eligibility criteria but is discussed in Section 6 because it bears directly on the interpretation of the HMO analyses.

Titles and abstracts were screened for relevance by the authors, and the full texts of potentially eligible records were assessed jointly; disagreements were resolved by discussion. Studies were considered directly relevant when they measured, supplied or manipulated milk oligosaccharides and assessed an oral organism, an oral surface model or the oral microbiome.

Given the small number of directly relevant human studies, mechanistic and comparative material from the intestinal literature is incorporated where such extrapolation is explicitly signposted. Where evidence is absent rather than merely limited, this is stated.

Because the evidence is heterogeneous, each mechanistic claim is classified by the type of evidence supporting it rather than graded for strength, using four descriptive categories: (i) human oral evidence, meaning associations observed in infants, which are observational unless stated otherwise and do not establish causation; (ii) direct oral in vitro or ex vivo evidence, obtained with oral organisms or oral surface models; (iii) indirect evidence from other anatomical compartments or from non-oral organisms; and (iv) unresolved hypothesis, where no direct evidence was identified. These categories do not constitute a formal quality appraisal. Funding sources and declared conflicts of interest of the principal studies are reported in the notes to Table 1 and Table 2.

## 3. Human Milk Oligosaccharides: Structure and Maternal Determinants

### 3.1. Structural Principles

Every HMO is built on a lactose disaccharide at the reducing end. Elongation proceeds through the addition of lacto-*N*-biose or *N*-acetyllactosamine units, and the resulting backbone—linear or branched—is decorated by fucose in α1-2, α1-3 or α1-4 linkage, or by Neu5Ac in α2-3 or α2-6 linkage [1,3]. From this limited grammar arise more than two hundred characterized structures, of which perhaps fifteen account for the bulk of the molar mass in any individual sample; in the BLOSOM cohort the most abundant were 2′-fucosyllactose, difucosyllacto-*N*-tetraose, 3-fucosyllactose, lacto-*N*-fucopentaose II and lacto-*N*-tetraose [2]. Neutral fucosylated and neutral non-fucosylated species together account for the great majority of the pool, and acidic sialylated species for a minor fraction [1]. In the milk of secretor mothers, 2′-fucosyllactose is the single most abundant structure [35].

The distinction between neutral fucosylated, neutral non-fucosylated and acidic sialylated species is not merely descriptive. Charge and terminal residue determine which host and bacterial lectins a given oligosaccharide engages. Even within a single fucosylated species, assimilation strategies differ by bacterial strain: *B. longum* subsp. *infantis* isolates handle 2′-fucosyllactose, 3-fucosyllactose and difucosyllactose through distinct routes [36]. Speaking of “HMOs” as a functional class, in the oral compartment or elsewhere, obscures more than it clarifies.

### 3.2. Maternal Secretor Status and the FUT2 Polymorphism

The single largest source of between-mother variation is the FUT2 gene, which encodes α1,2-fucosyltransferase and controls the presence of water-soluble blood group antigens in secretions [37,38]. Women carrying two inactivating alleles—non-secretors—produce milk essentially devoid of α1-2 fucosylated oligosaccharides, chiefly 2′-fucosyllactose but also lactodifucotetraose, lacto-*N*-difucohexaose I and lacto-*N*-fucopentaose I [37]. In secretors these species account for roughly a quarter to a half of total HMO content [38].

The frequency of the non-secretor phenotype varies geographically. Non-secretors account for approximately one fifth of the United States population, and α1,2-fucosylated species generally represent 25–50% of total HMO content in the milk of secretor women [38]. In the German LIFE Child cohort, milk from 156 mothers at three months, 122 at six and 28 at twelve was genotyped and profiled for 24 HMOs: the nonsense variant rs601338 defined a non-secretor proportion of 11.8%, with strong correlations between secretor status and the concentrations of both 2′-fucosyllactose and lacto-*N*-fucopentaose I [35]. The abundance of 2′-fucosyllactose has accordingly been used as a proxy for genotyping, including in the BLOSOM analyses [2,37]. Population-specific variants complicate this further: in Chinese cohorts the relevant polymorphism is rs1047781 rather than rs601338, with corresponding differences in the milk glycobiome and in its effects on infant gut colonization [39].

The loss is partially compensated. Milk from non-secretor mothers is redistributed rather than simply depleted, with higher relative abundances of lacto-*N*-tetraose, lacto-*N*-fucopentaose II and III, and lacto-*N*-difucohexaose II [37,40]. Whether this redistribution is functionally equivalent remains unresolved, and the question acquires force in the oral context, since lacto-*N*-fucopentaose III was one of the two oligosaccharides inversely associated with infant oral bacterial richness [2].

A second fucosyltransferase, FUT3, catalyzes α1,3/4 transfer and defines Lewis status; secretor and Lewis status together, along with gestational age, generate correspondingly distinct milk glycan profiles [25,35,40]. Around 70% of populations studied are Lewis-positive secretors and some 5–10% Lewis-negative secretors [25].

### 3.3. Lactation Stage and Other Maternal Variables

HMO output is not static. Total concentration falls from colostrum to mature milk, and the profile shifts in parallel: 2′-fucosyllactose and lacto-*N*-tetraose reach their maxima in early lactation and decline thereafter, whereas 3-fucosyllactose rises across the later months [35]. Longitudinal work in other cohorts confirms that HMO profiles, milk microbiota and infant gut microbiota shift together with maternal characteristics over the course of lactation [41,42].

Beyond genotype and lactational stage, reported determinants include parity, mode of delivery, maternal age, diet and nutritional status, adiposity, geographic location and season [14,43,44]. Effect sizes attributed to these variables are modest relative to FUT2, and the literature is heterogeneous enough that individual associations warrant reserve.

Dose is a separate matter from concentration. Milk intake varies substantially between infants and across the day [45], though it remains broadly stable between one and six months [46], with the consequence that two infants receiving milk of identical composition may nonetheless receive markedly different daily quantities of any given oligosaccharide. The BLOSOM investigators made this distinction operational by modeling concentration and 24 h intake separately, and obtained partly divergent results [2]—a methodological point of some importance, considered again in Section 5.1.

### 3.4. HMOs and the Milk Microbiota

Human milk is not sterile, and its bacterial content covaries with its glycan content. Maternal secretor status has been associated with milk microbiota composition [47], and integrated analyses in larger cohorts have related HMO profiles to both milk bacterial communities and milk fatty acids [48], as well as demonstrating shifts between colostrum and mature milk [42]. The relevance here is direct: the infant oral cavity receives both the glycans and the bacteria in the same bolus, so any oral effect attributed to HMOs must in principle be distinguished from an effect of the milk bacteria whose abundance those same HMOs help determine. Neither of the two available oral studies was designed to make that separation.

### 3.5. Why FUT2 Matters Twice in the Oral Compartment

One consequence of the FUT2 polymorphism has been largely absent from discussions of infant oral health. The same gene, expressed from the infant’s own alleles rather than the mother’s, governs α1,2-fucosylation of glycans in the infant’s secretions; non-secretor individuals lack the major α1,2-fucosylated glycans in body secretions, saliva included [35]. The salivary films to which streptococci adhere—the acquired enamel pellicle once teeth have erupted, and the related but compositionally distinct mucosal pellicle before and after eruption—are therefore likely to be patterned by the infant’s own secretor status, independently of the mother’s. Because one *FUT2* allele is inherited from the mother, the two genotypes are correlated but not identical.

The oral cavity therefore presents a two-locus problem that the intestinal literature does not pose in the same form. Maternal FUT2 governs the glycan cargo delivered with each feed; infant FUT2 governs the host glycans those molecules encounter. Maternal secretor status has been incorporated into the existing oral analyses—as a stratifying variable in the smaller study [49] and as a model covariate in the larger [2]—but in both it was inferred phenotypically from 2′-fucosyllactose abundance rather than genotyped, and infant FUT2 status has not been assessed in any infant oral microbiome study we identified. A caveat belongs here: whatever the secretor phenotype, every lactating woman synthesizes the core HMO repertoire, so inter-individual variation should not be read as functional deficiency [35,40]. What varies is the balance of structures, and it is the balance rather than the presence of glycans that the argument below turns on. In the intestine, infant secretor phenotype has been reported to associate more strongly with fecal community structure than maternal genotype does [38], which at minimum establishes that the distinction is worth drawing.

## 4. The Infant Oral Microbiome: Composition and Temporal Development

### 4.1. Composition

Any account of how HMOs might shape oral colonization requires a description of what is being shaped. The most detailed comes from the BLOSOM cohort, in which 667 oral samples were collected at ten timepoints from the first week to the second year of life in 84 predominantly breastfed infants and profiled by full-length 16S rRNA gene sequencing [50]. Three taxa dominated: *Streptococcus mitis*, *Gemella haemolysans* and *Rothia mucilaginosa* [50]. In the cross-sectional analysis at two months, *S. mitis* alone accounted for a mean relative abundance of 34.5%, with *Streptococcus salivarius* group 1 at 8.5% [2]. Comparable dominance has been described in independent cohorts [51,52,53,54]. This is an aerotolerant, facultatively anaerobic assemblage, and it bears little resemblance to the bifidobacterial community that HMOs are held to select for downstream.

### 4.2. Temporal Development

Development is neither linear nor monotonic. Bacterial richness fell between one and two months and rose again between twelve and twenty-four months, while Shannon diversity increased from the first week to the first month and again across the intervals from six to nine and nine to twelve months [50]. The trajectory matters for interpretation: a cross-sectional association between an HMO concentration and diversity acquires different meanings depending on where in that trajectory sampling occurred.

Acquisition nonetheless follows an organized pattern rather than a random one [51], and the succession across childhood is influenced by postnatal factors and associated with subsequent tooth decay [55]. The maternal contribution is substantial: salivary communities of edentulous infants resemble those of their mothers or primary caregivers [54], and systematic review of maternal and neonatal oral developmental patterns supports a strong familial signal [56].

### 4.3. Feeding Mode

Feeding mode is among the strongest discriminants. Oral microbial profiles separate breastfed from formula-fed infants [15], and both community composition and developmental pattern differ by feeding practice [16,17]. Within breastfed infants the relationship appears dose-dependent: higher total milk intake was associated with lower relative abundance of *Streptococcus parasanguinis* in the same cohort [57], an effect the authors suggest may be partly HMO-mediated. The broader set of early-life determinants has been reviewed by the same group [58], and includes pacifier use, intrapartum antibiotic prophylaxis, mode of delivery, maternal allergy, pre-pregnancy body mass index and the presence of siblings [50].

### 4.4. Streptococcus mutans and the Timing Problem

*Streptococcus mutans* warrants separate mention, since it is the organism around which most oral-health interest in infant feeding revolves, and because feeding mode appears to affect it directly. Córdova-Carrillo et al. cultured saliva from 30 infants under six months on Mitis Salivarius agar and found mean colony counts an order of magnitude lower in the exclusively breastfed group than in the formula-fed group, with mixed feeding intermediate [59]. The study is small, single-center, and culture-based rather than molecular, and 90% of parents reported performing no oral hygiene at all, so the comparison rests on feeding practice within a uniformly unbrushed population. Streptococci more generally are pivotal early colonizers, shaping biofilm formation and subsequent microbial succession [18]. Early acquisition also sets a timing constraint on any nutritional argument: by the time HMO exposure is typically measured, the organism of greatest clinical interest may already have arrived, so any HMO effect operates on a community that is already partly determined. Timing also determines which surfaces are available. The infants sampled in the HMO analyses were two to four months old and therefore pre-dentate, so colonization surfaces were mucosal; adhesion to the acquired enamel pellicle, the surface used in most *S. mutans* adhesion experiments, becomes relevant only after tooth eruption.

**Table 1:** Human studies relating HMO exposure to the infant oral microbiome.

Study	Design and Sample	HMO Assessment	Principal Oral Findings
Cheema et al., 2022 [49]	Longitudinal; 10 exclusively breastfed dyads; maternal fecal and milk, infant oral and infant fecal sampling to 4 months	19 HMOs by HPLC; concentrations and calculated daily intakes	Multiple associations varying by timepoint and by maternal secretor status; DFLac intake associated with *S. mitis*
Arishi et al., 2025 [2]	Cross-sectional at 2 months; 56 dyads (54 exclusive, 2 predominant); full-length 16S rRNA sequencing	19 HMOs by HPLC; concentrations and 24 h intakes; secretor status inferred from 2′FL	3FL inversely associated with Shannon diversity; LNFP III with richness; LNFP II with *Neisseria subflava*; DFLNT and DFLNH intakes with *S. parasanguinis*. Effects characterized by the authors as biologically limited
Arishi et al., 2026 [34] (no HMOs measured; shown for context)	Cross-sectional at 3 months; 45 exclusively breastfed dyads; full-length 16S rRNA sequencing	None; lactose, lactoferrin, lysozyme, secretory immunoglobulin A and 12 minerals; concentrations and estimated 24 h intakes	Lactoferrin concentration and intake inversely associated with Shannon diversity; lysozyme and secretory IgA associated with specific taxa, including *Gemella haemolysans*; lactose associated with no oral feature; concentration effects stronger than intake effects, partly a possible mathematical artefact

2′FL, 2′-fucosyllactose; 3FL, 3-fucosyllactose; DFLac, difucosyllactose; DFLNH, difucosyllacto-*N*-hexaose; DFLNT, difucosyllacto-*N*-tetraose; HPLC, high-performance liquid chromatography; LNFP, lacto-*N*-fucopentaose. All three analyses derive from the BLOSOM cohort (85 dyads in total); the overlap between their samples was not reported and is likely. Funding and conflicts of interest: Refs. [2,34] declare an unrestricted research grant from Medela AG, administered by The University of Western Australia and participation of one author in the Medela AG Scientific Advisory Board; Ref. [49] derives from the same cohort and research program.

## 5. Mechanisms by Which HMOs May Act in the Oral Cavity

One interpretive constraint applies to all the mechanisms considered below. An association between a milk oligosaccharide and an oral taxon is not, on its own, evidence of substrate use or receptor-mediated action. Individual HMOs correlate strongly with one another because they share biosynthetic pathways [2]; maternal secretor status alters other glycosylated secretions as well as milk [35]; the milk microbiota covaries with the HMO profile [47,48]; and milk volume and feeding frequency differ between infants [57]. Other milk components act on the same community: in a further BLOSOM analysis that did not measure HMOs, lactoferrin concentration and intake were both inversely associated with infant oral Shannon diversity [34], so an HMO–diversity association could partly reflect co-varying proteins. Delivery mode, siblings and pacifier use also shape the infant oral community independently of milk composition [50]. The mechanisms below are therefore candidates, and their plausibility is assessed separately from the associations reported in Section 6.

### 5.1. Physiological Differences Between the Oral and Intestinal Compartments

**Figure 1:**
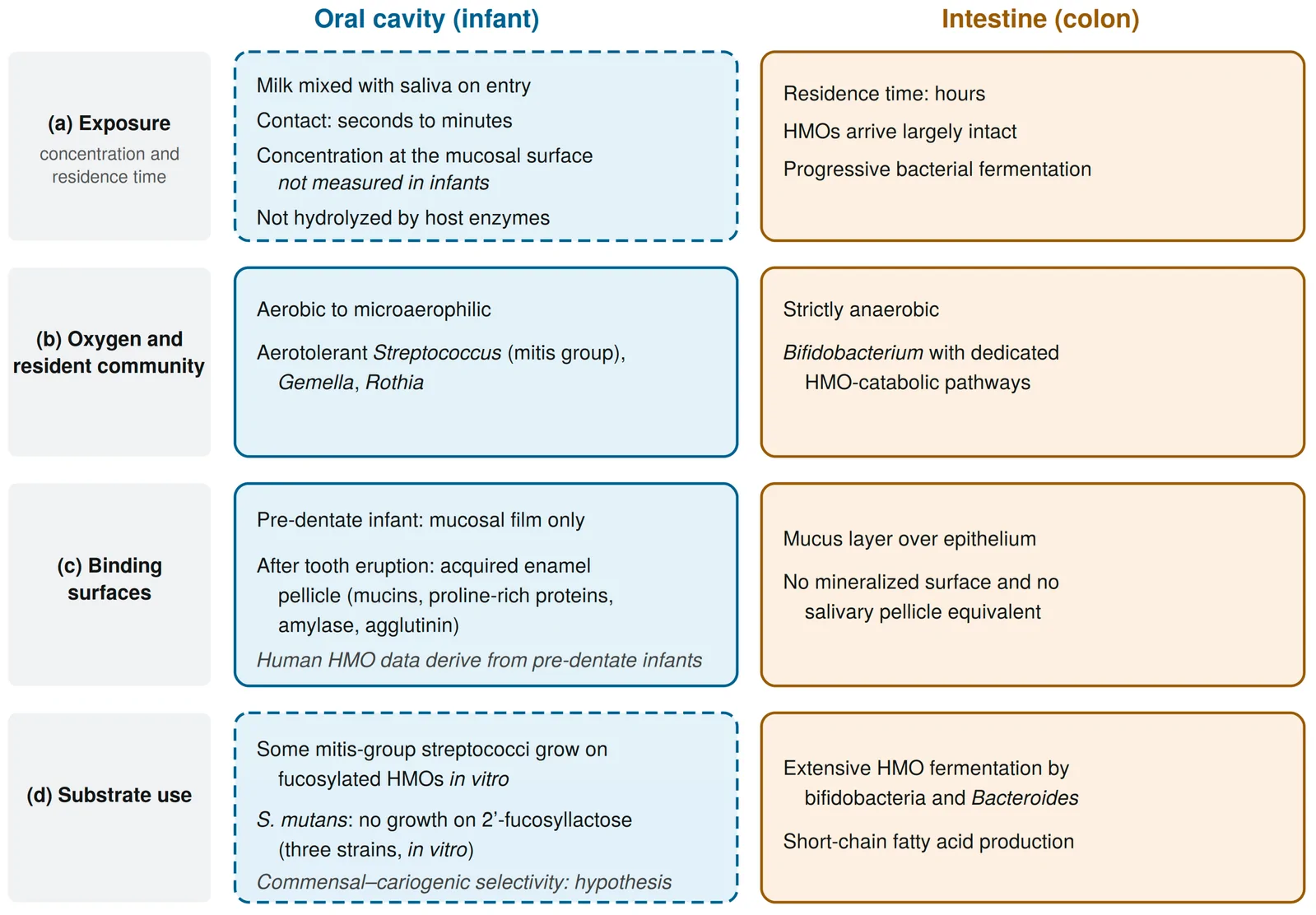
Oral and intestinal compartments as sites of HMO exposure. (**a**) Exposure: in the mouth, milk is mixed with saliva, contact lasts seconds to minutes and the effective surface concentration has not been measured; in the colon, residence lasts hours. (**b**) Oxygen tension and resident communities: aerotolerant streptococci, *Gemella* and *Rothia* in the mouth; strictly anaerobic bifidobacteria with dedicated HMO-catabolic pathways in the colon. (**c**) Binding surfaces: mucosal pellicle in the pre-dentate infant and acquired enamel pellicle after tooth eruption, with no equivalent in the colon. (**d**) Substrate use: fucosylated HMOs support the growth of some mitis-group streptococci in vitro but not of *Streptococcus mutans*. Dashed outlines indicate features inferred rather than measured in infants.

Three features distinguish the oral compartment from the intestinal one, and each qualifies a different element of the prebiotic model. A fourth commonly assumed difference does not survive scrutiny, and is addressed in Section 5.4. These differences are summarized in Figure 1.

Residence time differs most clearly, and concentration probably differs too. HMOs enter the mouth at the concentration present in milk [1,2] but are immediately mixed with saliva; their effective concentration at mucosal or dental surfaces depends on milk flow, salivary flow, swallowing, surface adsorption and clearance, and we identified no study that measured it during or after a feed. They do arrive enzymatically intact. Gnoth et al. incubated neutral and acidic HMO fractions from six milk samples with human salivary amylase, porcine pancreatic amylase and intestinal brush-border membrane vesicles, and neither amylase cleaved them; only prolonged exposure to the vesicles produced slight modification [4]. Resistance to hydrolysis in the upper gastrointestinal tract has been confirmed independently [5]. Whatever these molecules do in the mouth, host digestion is not part of it. Contact, however, lasts seconds to minutes rather than hours. The oral cavity is therefore plausibly, though not demonstrably, a site of higher-concentration and shorter-duration exposure than the colon. Arishi et al. drew a related distinction in proposing that HMO concentration may act locally on adhesion during feeding while total daily intake reflects systemic effects, and reported divergent associations for the two measures [2]. A later analysis from the same cohort, examining lactose, antimicrobial proteins and minerals rather than HMOs, found considerably stronger effects for milk concentrations than for estimated intakes, while noting that intake is calculated from concentration and that the difference may partly be a mathematical artefact [34].

Oxygen tension shapes the second difference. The community described above is dominated by aerotolerant streptococci, Gemella and Rothia [2,50], not by the strictly anaerobic bifidobacteria whose glycan-foraging machinery underpins the intestinal prebiotic effect [6,7]. The enzymatic solutions those organisms evolved for HMO catabolism, including the lacto-*N*-biose pathway, have no straightforward counterpart in the oral streptococcal repertoire.

Third, the mouth presents a surface the intestine does not. Salivary proteins adsorbed onto oral surfaces supply the receptors through which streptococci attach [18,60], but two such surfaces should be distinguished. The acquired enamel pellicle, a proteinaceous film of mucins, proline-rich proteins, α-amylase and agglutinin, forms on erupted teeth [61]; epithelial surfaces carry their own salivary film. In the pre-dentate infants sampled in the available human studies, only mucosal surfaces were present. Adhesion is a necessary early step in biofilm establishment, although growth, carbohydrate metabolism, extracellular polysaccharide production, acid tolerance, competition between species and salivary clearance all shape what follows. A soluble glycan pool reaching these surfaces could, in principle, compete for bacterial binding sites.

### 5.2. Anti-Adhesive and Receptor Decoy Effects

The oldest relevant evidence requires careful reading, because it cuts against the glycan hypothesis rather than for it. Danielsson Niemi et al. fractionated human milk and screened purified constituents for inhibition of *Streptococcus mutans* binding to saliva- and gp340-coated hydroxyapatite, a system designed to reproduce the pellicle-covered enamel surface [31]. Avid inhibition was obtained with caseins, lactoferrin, IgA and IgG, and moderate inhibition with α-lactalbumin and bile salt-stimulated lipase; the inhibitory epitope of β-casein was localized to a defined C-terminal peptide [31]. Every compound identified was a protein. Allison et al. later reported that whole human milk increases *S. mutans* biofilm formation, with casein increasing it, lactoferrin and IgA decreasing it, and lactose without effect [32]—again a protein-dominated account.

Neither study assayed free oligosaccharides. The anti-adhesive activity documented was therefore attributable to its protein fraction; glycan involvement remains conjectural. Most discussions of breastfeeding and caries overlook this distinction. The receptor-decoy principle is nonetheless well established for milk oligosaccharides at other mucosal surfaces, where human and bovine species inhibit Neisseria meningitidis pilus attachment [30], and where HMOs restrict the growth of group B Streptococcus [29]. Anti-adhesive activity against intestinal pathogens is a recognized component of HMO function more broadly [12,13].

The distinction is not academic. If anti-adhesive activity at the enamel surface resides principally in the protein fraction, donor milk processing and formula supplementation carry quite different implications from those that follow if free oligosaccharides carry the effect—a point developed in Section 8.2.

### 5.3. Structure Dependence: Sialylated Versus Fucosylated Species

The most direct evidence concerns biofilm. Sato et al. cultivated *S. mutans* and mixed Streptococcus species under CO_2_ and evaluated 2′-fucosyllactose, 3′-sialyllactose, 6′-sialyllactose and Neu5Ac, quantifying biofilm by crystal violet assay, visualizing architecture by confocal laser-scanning microscopy with SYTO9/propidium iodide staining, and profiling transcriptomic responses by RNA-Seq [28]. Sialyllactoses and Neu5Ac inhibited biofilm formation more effectively than xylitol, with 6′-sialyllactose more effective than 3′-sialyllactose, and 2′-fucosyllactose producing no appreciable inhibition [28]. The proposed mechanism for the sialylated species is competitive masking of Neu5Ac glycan-binding sites [28].

Two caveats attach to this work and should be stated in any citation of it. The inhibitory concentrations were of the order of 100 mM, far above concentrations plausibly reached at oral surfaces during a feed, so the finding establishes a mechanism rather than a physiological effect. And it was performed with laboratory biofilm models rather than with communities from the infant mouth. Funding and affiliations of this and the other studies in this section are listed in the notes to Table 2.

We identified one study providing direct evidence on an individual HMO against an oral pathogen. Salli et al. tested 2′-fucosyllactose against three *S. mutans* strains—the type strain DSM 20523, Ingbritt and the clinical isolate CI 2366—assessing planktonic growth, adhesion to parotid saliva-coated hydroxyapatite by scintillation counting, and exopolysaccharide-mediated adhesion to glass, with galacto-oligosaccharides, xylitol, lactose and glucose as comparators [62]. Two results matter here. None of the three strains grew on 2′-fucosyllactose as a sole carbon source, while all grew on galacto-oligosaccharides, lactose and glucose [62]. And adhesion was reduced rather than enhanced: 2′-fucosyllactose diminished binding of the clinical isolate to saliva-coated hydroxyapatite and exopolysaccharide-mediated adhesion of the type strain, with no effect on Ingbritt [62]. The authors conclude that 2′-fucosyllactose, being non-fermentable by *S. mutans*, may lower acid production in plaque and does not promote adhesion. Only three strains were tested, and the concentrations exceeded those used in infant formula (funding: see notes to Table 2).

Further evidence, from non-oral panels, is consistent. Jarzynka et al. tested total and fractionated HMOs against four Gram-negative and three Gram-positive pathogens, reported predominant activity against the Gram-positive species, and explicitly excluded fucosyllactose as the antibacterial component [63]. The panel contained no oral commensal, so the result cannot be transferred to the oral compartment without qualification; what it establishes is narrower—that the antibiofilm activity of a whole-HMO preparation does not reside in its fucosylated fraction. Comparable structure-dependent antimicrobial and antibiofilm effects have been described against group B Streptococcus, *Staphylococcus aureus* and *Acinetobacter baumannii* [64,65]. Craft et al., testing fucosylated against non-fucosylated species in group B streptococcal systems, concluded that the presence of fucose does not by itself predict activity: position and degree of fucosylation determine it [66]. Native 2′-fucosyllactose lacked substantial antibiofilm activity in that system, whereas a cationic derivative, 1-amino-2′-fucosyllactose, reduced biofilm production by 46% and 37% in two strains [67]. Older data on human milk and its fractions likewise found effects on *S. mutans* biofilm formation that varied by component [32].

### 5.4. Fucosylated HMOs as Substrate: A Correction to the Prebiotic Objection

It would be convenient to argue that oral commensals lack the enzymatic repertoire to depolymerize complex oligosaccharides, and thus that fermentative selection cannot occur in the mouth. The evidence does not support the general form of that claim, though it does support a species-specific version of it that turns out to be more interesting. Streptococcus species possess fucosidase genes, and *S. mitis* grows more rapidly in vitro in media supplemented with 2′-fucosyllactose or lacto-*N*-fucopentaose I [68]. That demonstration comes from work on the milk metagenome rather than the oral cavity: Seferovic et al. found the majority of fucosidase-carrying species in human milk to be streptococci, including *S. mitis*, *S. pneumoniae* and *S. oralis*, and showed a carrying-capacity advantage for *S. mitis* and *S. oralis* in HMO-supplemented culture, using strains not necessarily isolated from milk [68]. Streptococci isolated directly from human milk have since been shown to consume 2′-fucosyllactose, 6′-sialyllactose and lacto-*N*-tetraose at rates comparable to *B. longum* subsp. *infantis* ATCC 15697 [69]. Members of the genus Neisseria likewise carry broad glycan utilization repertoires and show marked tropism and persistence within oral niches [70].

A possible asymmetry lies within the genus. The commensal mitis-group organisms that dominate the breastfed infant mouth appear able to use fucosylated HMOs; the cariogenic *S. mutans* cannot [62]. Whether this reflects the absence of the required α1,2-fucosidase, the transport machinery, or both is unresolved, though the genome of *S. mutans* UA159 is known to encode four ABC transporters whose transcription under fucose or HMO exposure has not been examined [62]. If the pattern holds across strains, HMOs would occupy an unusual position among milk carbohydrates: a substrate that feeds the commensal early colonizers while withholding carbon from the principal cariogen. This remains a hypothesis: it rests on a small number of strains, most not isolated from the infant oral cavity, and on growth assays rather than community experiments, and the transport and fucosidase mechanisms involved are unresolved. Lactose and galacto-oligosaccharides do no such thing, since *S. mutans* ferments both [62].

Concentration complicates this. Working with pooled HMOs against mature biofilms, Jarzynka et al. observed that bactericidal activity disappeared above roughly 20 mg/mL, apparently because the oligosaccharides ceased to act as ligands and began to act as nutrients [63]. Read alongside the physiological range of mature milk, the implication is a non-monotonic dose–response: too little glycan and receptor occupancy is insufficient to disturb biofilm initiation, too much and the same molecules feed the organisms they were inhibiting, with the window of maximal activity falling between [63]. Whether an equivalent inversion occurs at the pellicle, where concentrations during a feed have not been measured, has not been tested. It would be worth testing, since it bears directly on whether HMO-supplemented formula delivers glycan to the mouth in the inhibitory or the nutritive part of the curve.

The human associations are compatible with this reading, although they cannot establish it. Daily intakes of difucosyllacto-*N*-tetraose and difucosyllacto-*N*-hexaose were positively associated with *S. parasanguinis*, and difucosyllactose intake with *S. mitis* in the earlier study—a consistent affinity of oral streptococci for structurally related fucosylated oligosaccharides, plausibly reflecting shared adhesin binding motifs or shared substrate utilization [2,49]. Lacto-*N*-fucopentaose II, meanwhile, was associated with higher abundance of Neisseria subflava [2].

It would be tidy to assign the two fractions to two mechanisms—sialylated against adhesion and biofilm, fucosylated as substrate—but the evidence does not partition that way. Fucosylated 2′-fucosyllactose reduces *S. mutans* adhesion in two of three strains tested [62], so it is not purely nutritional; milk-derived streptococci consume 6′-sialyllactose as readily as 2′-fucosyllactose [69], so the sialylated fraction is not purely inhibitory. The evidence supports a narrower proposition: that individual HMOs act through several mechanisms at once, that the balance differs by structure and by bacterial species, and that the relevant axis may be commensal versus cariogenic rather than fucosylated versus sialylated. Seferovic et al. found no relationship between HMO-bound sialic acid and sialidase gene abundance, reading this as evidence that fucosylated species drive milk microbiome composition more strongly [68], which is at least consistent with the fucosylated fraction mattering most. Whichever reading holds, the fucosylated fraction is both the one most affected by maternal secretor status and the one added to commercial formula, which makes it a reasonable initial focus. Table 2 sets out the candidate mechanisms, classified by the type of evidence behind each (Section 2).

### 5.5. Immunomodulation at the Oral Mucosa

A third route bypasses bacteria altogether. HMOs modulate epithelial and immune cell function in the intestine and contribute to innate protection of the neonate more generally [10,11,12], and oropharyngeal-associated lymphoid tissue is the rationale underlying colostrum administration in neonatal units [71]. Salivary immunoglobulin A is itself a long-recognized determinant of oral microbial ecology [72]. Whether HMOs specifically, as distinct from secretory IgA and lactoferrin, contribute to that stimulation has not been established. Komatsu et al., surveying HMO effects by region of the gastrointestinal tract, treat the oral cavity as one such region and gather the available anti-adhesion and antibiofilm data on *S. mutans* and *Candida albicans* there [73]. On the epithelial side, Gürsoy et al. applied HMOs to an organotypic oral mucosal model and measured secretion of human β-defensins 2 and 3, hCAP18/LL-37 and proinflammatory cytokines [74]. That such a model exists, and has been used for precisely this question, weakens any claim that the oral immunological effects of HMOs are wholly unexplored; what remains unexplored is whether those effects translate into altered colonization in vivo. Arishi et al. raise the same possibility in proposing that daily HMO intake may act on the oral community indirectly, through host immune mechanisms influencing mucosal tolerance [2].

**Table 2:** Candidate mechanisms of HMO action in the oral compartment and the evidence supporting each.

Proposed Mechanism	Evidence Type	Supporting Observations	Level of Evidence (Section 2)
Receptor decoy/competitive inhibition of adhesion	In vitro, hydroxyapatite and mucosal models	Milk compounds reducing adhesion to ligand-coated hydroxyapatite were all proteins [31,32]; 2′FL reduced *S. mutans* adhesion in 2 of 3 strains [62]; inhibition of *N. meningitidis* pilus attachment [30]	(ii) Direct oral in vitro; activity largely in the protein fraction; glycan effect strain-dependent; no in vivo confirmation
Disruption of biofilm formation and architecture	In vitro, mono- and mixed-species biofilms	6′SL and Neu5Ac inhibit *S. mutans* biofilm more effectively than xylitol [28]; fucosyllactose excluded as the antibacterial component in a non-oral pathogen panel [63,64,65]	(ii) Direct oral in vitro, at supraphysiological concentrations (~100 mM); no in vivo confirmation
Substrate-driven selection of oral streptococci	In vitro growth data plus human association	Growth advantage for *S. mitis* and *S. oralis* [68]; milk isolates consume 2′FL, 6′SL and LNT near B. infantis rates [69]; but no *S. mutans* strain grows on 2′FL [62]	(ii)–(iii) In vitro growth data, partly with non-oral isolates; (i) human association only; commensal–cariogenic selectivity is an unresolved hypothesis (iv)
Direct growth inhibition of specific taxa	In vitro	HMOs restrict group B Streptococcus growth [29,64]; activity predominantly against Gram-positive non-oral species [63,65]	(iii) Indirect, non-oral organisms; not tested against oral commensals
Mucosal immunomodulation	Extrapolated from intestinal and systemic work	HMOs regulate hBD-2, hBD-3, LL-37 and cytokines in an organotypic oral mucosal model [74]; salivary IgA shapes oral ecology [72]	(ii) Oral mucosal model; effect on colonization unresolved (iv)

Levels of evidence: (i) human oral evidence, observational associations unless stated otherwise; (ii) direct oral in vitro or ex vivo evidence; (iii) indirect evidence from other compartments or non-oral organisms; (iv) unresolved hypothesis. Funding and affiliations: Ref. [28] was conducted at a corporate research institute; Refs. [62,74] were conducted by investigators employed by a manufacturer of commercial 2′-fucosyllactose; the BLOSOM analyses [2,34,49] are supported by an unrestricted research grant from Medela AG (see notes to Table 1). 2′FL, 2′-fucosyllactose; 6′SL, 6′-sialyllactose; LNT, lacto-*N*-tetraose; Neu5Ac, *N*-acetylneuraminic acid.

## 6. Human Evidence: Two Analyses from One Cohort, Modest Associations

The empirical base can be stated in a few paragraphs.

When Arishi et al. analyzed 56 mother–infant dyads [2], only one earlier study had related HMO exposure to the infant oral microbiome, and it was based on ten dyads from the same cohort [49]. That earlier study profiled maternal fecal, human milk, infant oral and infant fecal samples longitudinally in exclusively breastfed dyads, quantifying nineteen HMOs by high-performance liquid chromatography (HPLC) and identifying multiple associations that varied across the first four months and differed by maternal secretor status [49]. The larger analysis was cross-sectional at two months, with models adjusted for mode of delivery, siblings, maternal pre-pregnancy body mass index, pacifier use in the first week and secretor status; milk concentrations of 3-fucosyllactose were inversely associated with infant oral Shannon diversity, and lacto-*N*-fucopentaose III with richness [2]. Both analyses draw on the BLOSOM cohort of 85 dyads [34]. Neither reports the extent to which their participants overlap, and overlap is likely, so they are best read as two analyses within one research program rather than as independent studies. Findings are summarized in Table 1.

The authors’ own summary deserves to be reproduced in substance rather than softened: the effect of HMOs on infant oral microbiome development was statistically significant but biologically limited [2]. They further note that many associations did not survive correction for multiple comparisons, that several HMOs showed variance inflation factors above ten owing to their shared biosynthetic pathways, and that post hoc calculations indicate reliable detection only of correlations of approximately r = 0.33–0.36 or larger [2]. The associations are best read as hypothesis-generating rather than as evidence of a major structuring influence.

Several things nonetheless argue against dismissing the question. The diversity associations run opposite to the naive intestinal expectation—higher concentrations of specific fucosylated species accompanied by lower diversity—which is more readily reconciled with selective enrichment of a few adapted taxa, or with selective inhibition of adhesion, than with generalized prebiotic stimulation. The direction of the streptococcal associations was consistent across the two analyses despite differences in design, sample size and analytical approach [2,49], although consistency within one cohort and one laboratory is not replication. And both studies measured HMO concentrations against 16S-defined community structure alone. Neither assessed adhesion, biofilm architecture, acidogenic output, or FUT2 genotype in either mother or infant.

A third analysis from the cohort did not measure HMOs but bears on the interpretation of the first two [34]. In 45 exclusively breastfed dyads sampled at three months, milk lactoferrin, lysozyme and secretory immunoglobulin A, together with several minerals, were associated with infant oral diversity or with specific taxa, whereas lactose was associated with no feature of the oral microbiome; the authors regarded the inverse association between lactoferrin and Shannon diversity as their most robust finding [34]. Two implications follow. Protein components that may co-vary with the HMO profile are themselves associated with the oral community, which widens the range of confounders any HMO association must contend with. And the principal milk carbohydrate showed no association, which does not support a general substrate effect of milk sugars on this community. That analysis applied a centered log-ratio transformation before differential abundance testing [34], an approach suited to compositional sequencing data.

A further limitation deserves emphasis because it is generic to the field rather than specific to these investigations. Infant oral samples are low-biomass, and low-biomass sequencing is vulnerable to reagent and environmental contamination. The BLOSOM analyses addressed this with decontaminated PCR reagents and reported negative extraction and template controls [2], which is more than many comparable studies do; nonetheless, the interpretive caution warranted for low-biomass work applies here in full.

## 7. The Oral Compartment as an Intermediate in Gut Colonization

Most of the intestinal HMO literature rests on an assumption: that milk glycans transit the mouth as a corridor, not a station, en route to the colon. The assumption is convenient but has not been tested.

Infants swallow their oral community continuously. If HMOs shape which taxa dominate the oral cavity, and if those taxa are then delivered to the stomach and small intestine in every swallow, then it is conceivable that part of what is attributed to direct HMO action in the gut is mediated by the oral compartment. No direct evidence currently supports this possibility, and it is offered here as a hypothesis. The proposition is not exotic: oral organisms have been recovered from distant sites in conditions ranging from ventilator-associated pneumonia [20] to inflammatory bowel disease, where oral Campylobacter concisus strains have been examined for enteric pathogenic potential [21]. Such findings, drawn from adult disease, show that oral organisms can reach distant sites; they do not show that HMO-mediated changes in the infant oral community influence gut colonization.

Evidence bearing directly on the infant case is scarce. The one study that sampled maternal fecal, milk, infant oral and infant fecal compartments in parallel found that HMO intakes associated with taxa in more than one compartment, and that the infant oral community at days 2–5 differed markedly from all later timepoints [49]. Whether the oral community functions as a source, a filter, or merely a co-varying bystander cannot be determined from ten dyads.

The design required is not complicated. Paired oral and fecal sampling at matched timepoints, with strain-level resolution sufficient to establish whether the same organisms appear in both compartments and in what temporal order, would test organismal continuity. Even shared strains would not, by themselves, show that oral HMO effects caused downstream gut colonization; temporal ordering and mechanistic evidence would still be required. Full-length 16S sequencing, already applied in these cohorts [2,50], approaches but does not reach that resolution; shotgun metagenomics would.

**Figure 2:**
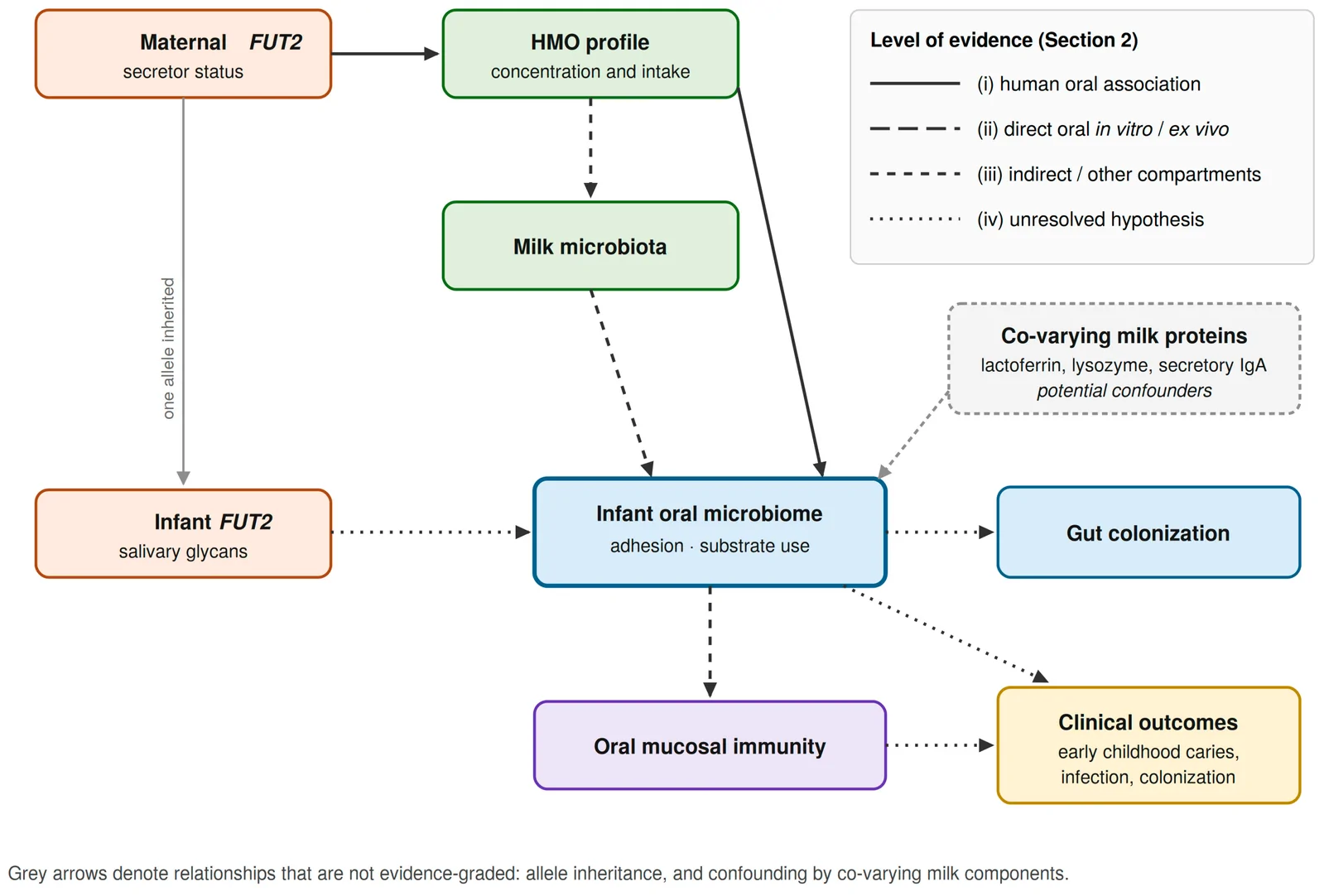
Proposed relationships linking maternal and infant *FUT2* genotype, human milk oligosaccharides, the milk microbiota, oral colonization, gut colonization, mucosal immunity and clinical outcomes. Line styles indicate the level of evidence for each link (Section 2): solid, human oral association (i); long dash, direct oral in vitro or ex vivo evidence (ii); short dash, indirect evidence from other compartments or organisms (iii); dotted, unresolved hypothesis (iv). Co-varying milk proteins, including lactoferrin, lysozyme and secretory immunoglobulin A [34], are shown as potential confounders of HMO–taxon associations.

The elements discussed separately in Section 3, Section 4, Section 5, Section 6 and Section 7 form an interconnected system rather than a sequence. Maternal and infant *FUT2* genotypes are correlated, because one allele is inherited from the mother; maternal secretor status shapes both the HMO profile and the milk microbiota; milk delivers glycans, bacteria and bioactive proteins together; and the oral and intestinal communities are linked by continuous swallowing. Figure 2 draws these relationships into a single scheme, with each link coded by the type of evidence supporting it, so that mechanistic plausibility is not mistaken for demonstrated infant physiology.

## 8. Translational Corollaries

### 8.1. HMO-Supplemented Infant Formula

Formulae containing 2′-fucosyllactose, alone or with lacto-*N*-neotetraose, are well tolerated, support normal growth, and have been evaluated in randomized multicenter trials [25,26]. Supplementation with 2′-fucosyllactose alongside galacto-oligosaccharides was associated with plasma inflammatory cytokine and TNF-α concentrations 29–83% lower than in infants receiving galacto-oligosaccharides alone, with no difference from breastfed infants [75]. The functional case for supplementation, on intestinal and immune grounds, is reasonably well made [11].

No trial has examined the oral microbiome as an outcome. Salli et al. framed their work around exactly this concern, comparing 2′-fucosyllactose against galacto-oligosaccharides because both are added to formula, and found that the latter supports *S. mutans* growth while the former does not [62]. Cariogenic potential cannot be inferred from the carbohydrate preferences of three *S. mutans* strains: lactose content, buffering capacity, feeding frequency, duration of tooth exposure, community structure and host factors all contribute. The observation warrants independent in vivo testing and does not, at present, support a preference between formula types on oral-health grounds. Formulations have meanwhile moved beyond the single-species products on which most trial evidence rests: blends of five HMOs—2′-fucosyllactose, difucosyllactose, lacto-*N*-tetraose and both sialyllactoses—have been evaluated in randomized trials and are now marketed [76]. Whether the sialylated components of those blends reproduce the antibiofilm effects seen in vitro is unknown. Adding oral sampling to an existing formula trial would impose a relatively low additional burden, although it still requires consent, standardized collection, storage, sequencing and contamination controls.

### 8.2. Donor Milk and Pasteurization

One property of HMOs is easy to overlook and consequential here: unlike the protein fraction, they withstand pasteurization and freeze-drying without appreciable structural degradation [77]. Their behavior during prolonged cold and heat storage has been less systematically documented. Donor human milk therefore delivers its oligosaccharide cargo substantially intact even where lactoferrin, secretory IgA and bile salt-stimulated lipase are compromised.

This dissociation is potentially informative, but it does not isolate a single variable. If the oral effects of human milk are glycan-mediated they should, in principle, survive Holder pasteurization, whereas glycoprotein-mediated effects should not; pasteurization, however, also inactivates the milk microbiota and alters other bioactive components. Comparisons between a mother’s own milk and pasteurized donor milk are further confounded by differences in maternal genotype, lactational stage, storage history and baseline composition. A within-mother comparison of raw and Holder-pasteurized own milk, with HMO profiles and protein bioactives quantified in both, would narrow these differences considerably, although it would still not separate glycan effects from the loss of milk-borne bacteria.

### 8.3. Oropharyngeal Colostrum in the Preterm Infant

Oropharyngeal colostrum administration is increasingly used in neonatal intensive care, on a rationale set out in theoretical terms before the supporting data existed [71]. The population concerned is one in which outcomes remain uneven between and within countries [78,79], and in which fungal as well as bacterial colonization of mucosal surfaces carries clinical weight [80]. A Cochrane review of randomized trials found the evidence for effects on mortality and major morbidity to be limited [81], while meta-analysis of very-low-birth-weight infants reported a reduction in ventilator-associated pneumonia [82]—a finding of some interest here, given the recognized traffic between oral and respiratory flora [20]. Maternal secretory IgA has separately been shown to protect against necrotizing enterocolitis in preterm infants [83].

The one study to examine the oral community directly was observational rather than randomized, following 20 preterm neonates weekly to day 21, half receiving the protocol and half standard care [27]. Cortez et al. reported higher relative abundance of Staphylococcus at day 7 in the protocol group and increased abundance of Bifidobacterium and Bacteroides, interpreting the pattern as accelerated maturation of the oral cavity [27]. Twenty infants cannot settle the question, and the direction of the Staphylococcus finding is not what an anti-adhesive account would predict. What the study does establish is that the intervention is measurable at the level of the oral community at all, which is the precondition for any mechanistic claim. Further randomized work is under way, including protocols with gut microbiota and metabolites as primary outcomes [84].

### 8.4. Breastfeeding and Early Childhood Caries

The literature here is conflicted, and this review states rather than resolves the division. A meta-analysis of twenty-five observational studies comprising 19,681 participants found an increased risk of early childhood caries in children breastfed beyond 24 months (RR 2.44; 95% CI 1.97–3.02), with no significant increase over the 12–24 month interval, and an odds ratio of 1.86 (95% CI 1.48–2.35) for caries prevalence beyond twelve months [85]. Earlier syntheses had reported a protective association for breastfeeding up to twelve months, with risk emerging thereafter [85,86,87].

Two readings are available and the data do not distinguish between them. Prolonged breastfeeding may be intrinsically cariogenic through nocturnal feeding and substrate availability at the tooth surface, or the association may be confounded by feeding practices, oral hygiene, socioeconomic position and dietary sugars, all of which cluster with extended breastfeeding in ways that observational designs handle poorly. The included studies used inconsistent definitions of early childhood caries and largely lacked prospective design or control for dental health practices [85]. Early microbial succession is itself associated with subsequent decay [55], which complicates any attempt to attribute risk to feeding practice alone.

What is missing from this debate is milk composition. No study in the caries literature has stratified by HMO profile or maternal secretor status, despite the fact that the composition of the fluid bathing the dentition differs substantially between mothers, and that roughly one woman in ten in a European cohort, and a higher proportion in some populations, produces milk devoid of α1-2 fucosylated species [35]. That caries risk among breastfed children might vary with maternal FUT2 status is an untested research hypothesis, and one that milk-bank or cohort archives could address retrospectively. The hypothesis concerns milk composition as a possible modifier of risk; it does not imply that HMO profiles explain the epidemiological association, and nothing in the present evidence argues against current recommendations to breastfeed.

### 8.5. Beyond the Mouth

Interest in the early oral community extends past dentistry. Oral organisms have been implicated, with varying degrees of evidential support, in periodontal disease and its systemic associations [18,24], atherosclerotic inflammation [22], rheumatoid arthritis [23], respiratory infection in ventilated patients [20] and gastrointestinal inflammation [21]; the general case for an oral contribution to systemic disease has been reviewed [19]. These associations are drawn overwhelmingly from adult populations, and extrapolating them to a two-month-old infant would be unwarranted. The relevance is more modest and more defensible: if early oral colonization has downstream consequences of any kind, then the nutritional determinants of that colonization acquire an interest they would not otherwise possess.

## 9. Clinical Implications and Public Health Perspectives

The evidence reviewed here does not justify changes to clinical practice on oral-health grounds, and we state this as a position rather than as a deferral to future research. Several practical conclusions nonetheless follow; they are summarized in Table 3.

For infant formula, current evidence does not support a preference between 2′-fucosyllactose-supplemented and galacto-oligosaccharide-supplemented products for the purpose of oral health. The relevant observation comes from a single manufacturer-funded in vitro study of three *S. mutans* strains [62], and no trial has measured an oral outcome, so the choice of formula should continue to rest on nutritional, tolerance and intestinal evidence [25,26]. Donor milk is a different case. Its oligosaccharide fraction is largely preserved through Holder pasteurization [77], so any glycan-mediated oral effect of human milk would be expected to persist while contributions from heat-labile proteins and milk-borne bacteria would not; this is compatible with the retention of potential HMO-related activity after pasteurization, but it does not demonstrate an oral microbiome benefit, and no clinical recommendation about donor milk follows from the evidence reviewed here.

Oropharyngeal colostrum in preterm infants remains justified by its neonatal rationale and by the trial evidence on clinical outcomes [81,82]. An effect on the oral microbial community has been reported in one small observational study [27] and should not be presented as an established benefit.

The association between breastfeeding beyond 12–24 months and early childhood caries is observational and plausibly confounded [85,86,87]. It does not justify discouraging breastfeeding. It does support oral hygiene counseling from eruption of the first tooth and attention to prolonged nocturnal feeding once teeth are present [88]. Whether maternal secretor status modifies caries risk is a research question without current clinical application.

From a public health perspective, the most useful immediate step is methodological: standardized oral sampling could be incorporated into trials and cohorts that already record milk, formula or colostrum exposure, where the additional burden is relatively low and the resulting data could test the hypotheses set out in this review.

## 10. Limitations and a Research Agenda

This review inherits the weaknesses of its evidence base. Two human analyses of HMO exposure, drawn from one cohort in one country and sharing most of their authorship, cannot support generalization; both are small, and the larger reported effects its own authors characterized as biologically limited [2]. The mechanistic material is almost entirely in vitro, much of it using monospecies biofilms and purified preparations at concentrations well above those achieved at the pellicle during a feed [28]. Extrapolation from intestinal work has been signposted where used, but remains extrapolation. Restriction to English-language publications may have excluded relevant work, particularly from pediatric dentistry studies published in other languages. As a narrative synthesis the present work carries no protocol registration and no formal appraisal of study quality.

The dependence on extrapolation deserves more weight than a single sentence. Much of the mechanistic framework rests on intestinal research and on in vitro systems whose conditions differ from those of the infant mouth in contact time, dilution by saliva, oxygen tension, the nature of the binding surface and salivary clearance (Section 5.1). Several key experiments used non-oral organisms, laboratory strains or concentrations well above plausible oral exposure, and an effect demonstrated only at supraphysiological concentration establishes biochemical possibility rather than an in vivo mechanism. The human data, for their part, are associations, vulnerable to collinearity among HMOs, to confounding by co-varying milk components and infant-level exposures, and to the compositional nature of relative-abundance data. These limitations do not weaken the oral-compartment hypothesis, but they restrict this review to generating and sharpening it; causal conclusions about the oral compartment itself cannot yet be drawn.

Five priorities follow. First, genotyping should be prioritized over phenotyping: secretor status in existing oral analyses was inferred from 2′-fucosyllactose abundance, and infant *FUT2* status has not been examined at all, despite the two-locus structure described in Section 3.5. Then there is the matter of function rather than taxonomy: 16S profiling cannot detect changes in adhesion, biofilm architecture or acidogenic output, which are the phenotypes that matter clinically; shotgun metagenomics, metabolomic assessment of salivary organic acids and ex vivo adhesion assays each address a different limitation. The sialylated fraction also deserves the attention that has so far gone to 2′-fucosyllactose, since it carries the demonstrated antibiofilm activity and has only recently entered supplemented formula. Paired oral and fecal sampling with strain-level resolution would establish whether the oral compartment mediates part of what is currently attributed to direct intestinal action. Finally, oral sampling should be appended to trials already in progress—supplemented formula studies, donor milk protocols and oropharyngeal colostrum trials—where the incremental burden is relatively low and the design work is already done.

A methodological point cuts across all five. Concentration and intake are not interchangeable exposures, and modeling them separately has already produced divergent results in the HMO analysis that attempted it [2], and a stronger effect of concentration than of intake has since been reported for other milk components [34]. Any future design that measures milk composition without measuring milk volume will remain unable to distinguish local from systemic effects.

Low-biomass oral samples also require specific safeguards. Future studies should report extraction blanks and no-template controls, include mock communities where appropriate, randomize samples across processing batches and describe contaminant-removal procedures transparently [89]. Because an increase in the relative abundance of one taxon may reflect a decrease in others, absolute bacterial load should be measured alongside sequencing, for example by quantitative PCR, and associations between individual HMOs and taxa should be tested with methods appropriate to compositional data, such as log-ratio transformation [90], with correction for multiple testing and explicit handling of collinearity between structurally related HMOs.

## 11. Conclusions

The oral cavity is the first compartment human milk oligosaccharides enter and among the least studied. Two analyses from a single cohort report statistically observed but modest associations between HMO exposure and the infant oral microbiome; several did not survive correction for multiple testing, and their direction does not fit a simple prebiotic model.

Mechanistic evidence is almost entirely in vitro. Sialylated species inhibit *S. mutans* biofilm formation, but at concentrations far above plausible oral exposure. Fucosylated species support the growth of some commensal streptococci but not of *S. mutans*, yet 2′-fucosyllactose also reduces *S. mutans* adhesion in some strains, and milk-derived streptococci consume sialylated as well as fucosylated structures. These findings do not partition cleanly by structural class. They are compatible with several mechanisms acting together, including interference with bacterial binding, species-selective substrate use and possibly mucosal immune effects, without establishing which of them, if any, operates in the infant mouth.

Current evidence does not justify changes to clinical practice on oral-health grounds, whether a preference between formula types or any revision of breastfeeding recommendations. The questions that would test the oral-compartment hypothesis are nonetheless well defined: whether maternal and infant *FUT2* genotypes jointly condition oral colonization, whether HMO-supplemented formula reproduces any oral association seen with human milk, whether glycan-mediated effects survive pasteurization, whether oral communities contribute to gut colonization, and whether caries risk varies with milk composition. Most can be addressed with existing methods, in several cases by adding oral sampling to studies already under way.

## Figures and Tables

**Table 3:** What current evidence does and does not support in clinical practice.

Setting	Current Evidence	What It Supports	What It Does Not Support
HMO-supplemented formula	One manufacturer-funded in vitro study of three *S. mutans* strains [62]; no trial with oral outcomes	Formula choice based on nutritional, tolerance and intestinal evidence [25,26]	A preference between 2′-fucosyllactose- and galacto-oligosaccharide-supplemented formula on oral-health grounds
Donor milk	HMOs largely preserved through Holder pasteurization [77]; no oral microbiome data	Retention of potential HMO-related activity after pasteurization	An oral microbiome benefit, or clinical recommendations about donor milk drawn from oral evidence
Oropharyngeal colostrum in preterm infants	Limited evidence on mortality and major morbidity [81]; reduced ventilator-associated pneumonia in meta-analysis [82]; one observational oral microbiome study (*n* = 20) [27]	Continued use on established neonatal indications	An established effect on the oral microbiome
Prolonged breastfeeding and early childhood caries	Observational association beyond 12–24 months, plausibly confounded [85,86,87]	Oral hygiene counseling from eruption of the first tooth; attention to nocturnal feeding once teeth are present [88]	Discouraging breastfeeding; attributing risk to HMO profile or maternal secretor status

## Data Availability

No new data were created or analyzed in this study. Data sharing is not applicable to this article.

## Abbreviations

The following abbreviations are used in this manuscript:

2′FL	2′-fucosyllactose
3FL	3-fucosyllactose
6′SL	6′-sialyllactose
DFLac	difucosyllactose
DFLNH	difucosyllacto-*N*-hexaose
DFLNT	difucosyllacto-*N*-tetraose
HMO	human milk oligosaccharide
HPLC	high-performance liquid chromatography
LNFP	lacto-*N*-fucopentaose
LNT	lacto-*N*-tetraose
Neu5Ac	*N*-acetylneuraminic acid
